# Three-dimensional image guidance for diagnosis and treatment of adrenal disease: a systematic review

**DOI:** 10.1007/s13304-025-02386-9

**Published:** 2025-10-09

**Authors:** Sofia Di Lorenzo, Farahdiba Zarin, Matteo Pavone, Didier Mutter, Marco Raffaelli, Michel Vix, Barbara Seeliger

**Affiliations:** 1 Division of Endocrine and Metabolic Surgery, Fondazione Policlinico Universitario Agostino Gemelli IRCCS, Rome, Italy; 2 Centro di Ricerca in Chirurgia delle Ghiandole Endocrine e dell’Obesità (CREO), Università Cattolica del Sacro Cuore, Rome, Italy; 3 Institute of Image-Guided Surgery, IHU Strasbourg, Strasbourg, France; 4 ICube, UMR7357, INSERM U1328 RODIN, University of Strasbourg, Strasbourg, France; 5 UOC Ginecologia Oncologica, Fondazione Policlinico Universitario A. Gemelli IRCCS, Catholic University of the Sacred Heart, Rome, Italy; 6 Research Institute Against Digestive Cancer, IRCAD, Strasbourg, France; 7 Department of Digestive and Endocrine Surgery, University Hospitals of Strasbourg, Strasbourg, France

**Keywords:** 3D reconstruction, Surgical planning, Intraoperative guidance, Image-guided surgery, Adrenal segmentation, Radiomics

## Abstract

Adrenal abnormalities are common imaging findings requiring comprehensive workup. Invasive diagnostic and therapeutic procedures, including adrenal surgery, demand expertise to mitigate risks of severe complications. Recent applications of 3D image analyses range from enhanced diagnosis to therapeutic guidance with anatomical visualisation. This study assesses the efficacy and clinical utility of 3D image analyses in managing adrenal disease. This systematic review, registered with PROSPERO (CRD42024500783), adheres to PRISMA guidelines. Searches of PubMed, Google Scholar and ClinicalTrials.gov up to August 2024 identified eligible studies. Extracted data included imaging modalities, 3D applications for diagnosis or treatment, volumetric segmentation techniques, and differentiation between pathologic and normal adrenal glands. Technological maturity was assessed with the IDEAL framework. Of 1813 studies screened, 30 met inclusion criteria, encompassing 5304 imaging exams (2060F/2106M, and 10 studies with 1115 cases), predominantly CT (27 CT, 90%; 2 MRI, 6.7%; 1 CT/MRI 3.3%). Volumetric reconstructions employed manual, semiautomatic and automatic methods for digital visualisation, and subsequent 3D printing. 3D analyses of normal vs. neoplastic adrenal tissue supported diagnosis in 18 studies (60%), and treatment in 12 (40%), improving understanding of respective anatomical features and planning for operative and interventional procedures. These applications represented early IDEAL stages of surgical innovation (0—preclinical, 1—idea and 2—development/exploration). 3D image analyses show promise in optimising adrenal disease management by aiding adrenal lesion characterisation and treatment optimisation through pre- and intraprocedural guidance. Further clinical assessment and long-term studies are warranted to establish the broader clinical impact.

**Graphical Abstract:**

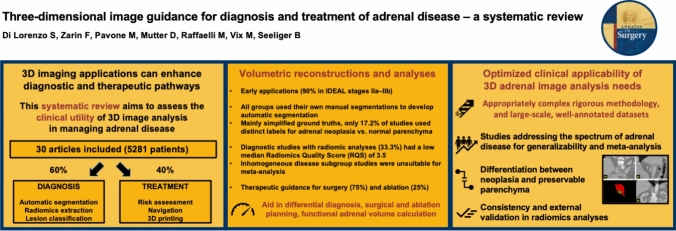

## Introduction

Adrenal abnormalities are increasingly detected due to widespread use of high-resolution cross-sectional imaging, with a prevalence of 10% in the elderly [1–3]. Determining the need for surveillance or surgery remains challenging, since various pathologies affect the adrenal glands. Comprehensive diagnostic workup involves clinical, genetic, hormonal and imaging studies that are costly, time-consuming and sometimes inconclusive [4]. Most incidentally discovered adrenal lesions are benign and non-functioning, but 2–3% are malignant. In oncology-based cohorts, malignancy rates can reach 30%, while larger clinical/surgical cohorts report adrenocortical carcinoma in up to 12% of cases [1, 5].

Surgical indications are primarily based on hormonal excess and malignancy suspicion, either partial or total adrenalectomy depending on the pathology and recurrence risk. In indeterminate lesions, where malignancy cannot be excluded, surgery is performed for diagnostic clarification. In 30–55% of procedures, histopathology was benign and would not have warranted adrenal surgery, reflecting a substantial overtreatment rate [1, 6, 7]. Often, total adrenalectomy is chosen over cortical-sparing approaches, resulting in unnecessary removal of functional adrenal tissue.

There is a clear need for more accurate non-invasive diagnostic tools to better characterise adrenal neoplasia preoperatively. Conventional radiology, relying on subjective visual assessments and linear measurements, may lead to diagnostic inconsistencies [8–10]. Radiomics, which extracts advanced quantitative image features, has emerged to address these limitations and enhance non-invasive diagnostic accuracy [11].

Three-dimensional (3D) patient-specific volumetric organ reconstructions derived from computed tomography (CT) or magnetic resonance imaging (MRI) improve anatomical visualisation to support planning and navigation for surgery and probe-guided ablation. Volume rendering (VR) enhances anatomical understanding but provides only a projected view. In contrast, volumetric segmentation masks including volumes of interest from surface rendering (SR), based on organ segmentation delineating all relevant structures, supports volume calculation, surgical simulation, and even 3D printing [12]. Manual segmentation is labour-intensive, and recent artificial intelligence (AI)-based approaches facilitate semi-automatic and automatic segmentation based on the use of large, validated data sets [13, 14].

3D imaging tools have wide-ranging applications in adrenal disease management, from diagnosis to post-treatment surveillance. Monitoring adrenal volume over time and employing deep-learning-assisted RECIST (response evaluation criteria in solid tumors) scoring can support lesion classification and growth assessment [15, 16]. In addition, texture analysis—a radiomics technique assessing intensity distributions and voxel relationships—holds promise for differential diagnosis support [17].

Augmented reality (AR), first applied in adrenalectomy in 2004, now encompasses mixed reality and telementoring, for remote collaboration, surgical training, and real-time intraoperative guidance [12, 13, 18]. While virtual 3D models enable intraoperative overlay, 3D-printed models support ex vivo training and practice. Patient-specific 3D reconstructions with precise anatomical segmentations are especially useful in complex oncologic and cortical-sparing adrenal surgery, offering tools for surgical navigation, planning, and quantitative imaging analyses.

However, a comprehensive literature synthesis is lacking to assess the role of 3D imaging, with and without artificial intelligence (AI) support, in minimising unnecessary surgery, preserving healthy tissue, and thus reducing morbidity. Therefore, this systematic review aims to analyse the clinical utility of 3D imaging in improving diagnostic accuracy, guiding treatment strategies, and enhancing surgical outcomes in adrenal disease.

## Methods

### Search strategy and study selection

The review was registered with the International Prospective Register of Systematic Reviews (PROSPERO, Registration N° CRD42024500783) and conducted according to Preferred Reporting Items for Systematic Reviews and Meta-Analyses (PRISMA) guidelines [19]. Articles were obtained by querying the databases PubMed, Google Scholar and ClinicalTrials.gov up to August 31, 2024. Relevant resources, references and online links were searched manually, and one article was added.

**Fig. 1:**
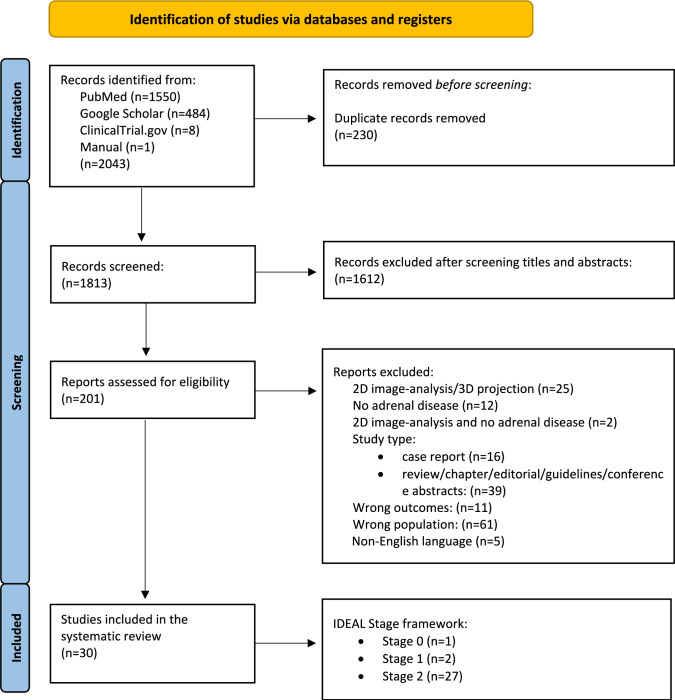
PRISMA flow diagram. Illustration of the study selection process with the following search terms: (3D printing OR 3D visualization OR image processing) AND (adrenal OR adrenalectomy OR paraganglioma)

According to the PICO/PEO (Participants, Intervention, Comparators or Controls, and Outcome) framework [20], only full-text articles were eligible that used volumetric reconstructions of the adrenal glands in patients with adrenal disease. Excluded were abstracts, reviews, meta-analyses, letters, case reports (< 3 cases) and editorials, as well as articles with 3D reconstructions for normal controls or patients without adrenal disease, studies on stereotactic/radiotherapy planning, studies on foetal ultrasound, preclinical and ex vivo studies, and non-English publications. The detailed study selection process is reported in the PRISMA flow diagram (Fig. 1). Rayyan software (Qatar Computing Research Institute, HBKU, Doha, Qatar)[21] was used independently by SDL and BS to screen titles and abstracts and remove duplicates, and all eligible full texts were independently reviewed by both authors. Technical questions related to semi-automatic and automatic segmentations were resolved through consultation with a third author specialised in computer science (FZ).

### Data extraction and analyses

For qualitative analysis, key features were extracted from the full texts of included studies, including author, country, year, study design (retrospective or prospective), sample size (number and sex of patients, number of imaging exams), and imaging modality. Information on the use of 3D imaging (for diagnosis or treatment), parenchyma and lesion segmentation volumes (if reported), and the study’s primary goal (e.g., differentiating lesions from normal adrenal tissue) was also collected.

Details on the type of 3D adrenal reconstruction (VR, SR, 3D printing) were recorded. Imaging analysis methods were noted, including segmentation strategy (manual, semiautomatic, or automatic), feature extraction software, and any machine learning application. Reported outcomes (positive or negative) were documented. In addition, each study was assigned an IDEAL stage to indicate the maturity and validation status of the technology [22, 23]. Two reviewers (SDL and MP) independently assessed risk of bias using the Quality Assessment of Diagnostic Accuracy Studies 2 (QUADAS-2) tool [24] classifying studies as having low, high, or unclear risk across four domains: patient selection, index test, reference standard, and flow and timing. The level of evidence was graded based on the Oxford Centre for Evidence-Based Medicine criteria [25].

For diagnostic studies using radiomics, the Radiomics Quality Score (RQS) was calculated (SDL and BS) to assess methodological quality, with 16 items of which the final score is converted to a percentage (with a score of 36 equalling 100%) [26].

Categorical data are reported as numbers and percentages, continuous variables as mean and standard deviation or median and interquartile range (IQR), depending on the distribution. All statistical analyses were performed using SPSS Statistics 25.0 software (IBM, Armonk, NY, USA).

## Results

### Study characteristics

Thirty studies published between 06/2004 and 08/2024 were included. A summary is provided according to the study aim in Tables 1 (diagnostic) and 2 (therapeutic). All but one study [27] were retrospective; one additionally incorporated a prospective validation cohort [28]. The studies covered 5281 patients (2060 female, 2106 male, and 1115 cases with unspecified sex across 10 studies). In total, 5304 imaging assessments were conducted, mostly CT-based (27 studies, 90%), including one combined with (11)C-metomidate PET [29]. Two studies used MRI (6.7%) [30, 31], and one (3.3%) both CT and MRI [32]. Seven studies (23.3%) involved ≤ 15 patients [29, 33–36], including two case series with 5 and 3 patients, respectively[37, 38]. The Oxford evidence level was 3–4[25]. The costs for volumetric reconstructions were not specified in any of the studies, and one study reported 3D printing costs (Table 2).

**Table 1:** Detailed diagnostic study characteristics

Authors	Years	Country	Imaging modality	Patients	Female patients n (%)	Segmentation method	Software and models	Adrenal gland/tumour volume (ml)	Detailed objective	Metrics	Outcome	Cost per 3D reconstruction	IDEAL stage
Li et al	2024	China	CT	1165	431 (34%)	Manual (1 Label), Automatic	ITK-SNAP nnU-Net	\	Normal vs. hyperplasia vs. nodule	On external validation set: DSC 0.88 ± 0.06, ICC 0.931 RVE 0.11 ± 0.11, HD95 2.26 ± 4.51;	Positive	N/A	2b
Chen et al	2024	Taiwan	CT	158	77 (48.7%)	Manual (1 Label)	3D slicer PyRadiomics (Radiomics)	UPA 6.77 ± 4.62 (L) 5.27 ± 3.58 (R) BPA 5.79 ± 3.07 (L) 4.89 ± 2.50 (R)	Subtype prediction (UPA vs. BPA); lateralization of PA	Accuracy 77.5 ± 3.9% Sensitivity 69.4 ± 6.7% Specificity 85.7 ± 2.7% F1 score 70.5 ± 7.1% AUC 0.771 ± 0.046	Positive	N/A	2a
Robinson-Weiss et al	2023	USA	CT	1242 (1265 imaging exams)	711 (57.2%)	Manual (1 Label), Automatic	nnU-Net Dense Net	**\**	Normal vs. abnormal	Segmentation, development test set: Median model DSC (normal glands) 0.80 (IQR, 0.78–0.89) (adrenal masses) 0.84 (IQR, 0.79–0.90) Classification, secondary test set: Overall sensitivity 69% (95% CI 58, 79) Overall specificity 91% (95% CI 90, 92)	Positive	N/A	2a
Xiao et al	2023	China	CT	134	73 (54.4%)	Semi-automatic (1 Label)	3D slicer PyRadiomics (Radiomics)	\	LPAA vs. PHEO	On external validation test (MLP model): Sensitivity 0.964 Specificity 0.962 AUC 0.979	Positive	N/A	2a
Wang L et al	2023	China	CT	182	\	Unspecified ground truth, Automatic (1 Label)	proposed combined encoder–transformer–decoder network	\	3D lesion mapping	DSC 0.858 Hausdorff distance 10.996 IOU 0.814 MAE 0.0005 ASD 0.509	Positive	N/A	2a
Singh et al	2023	USA	CT	91	45 (49.5%)	Manual (1 Label)	3D Densenet121 (Radiomics)	\	ACC vs. LPAA	Sensitivity-focused analysis: Accuracy 87.2% ± 8.13 Sensitivity 100% Accuracy-focused analysis: Accuracy 91% ± 0 Sensitivity 96%	Positive	N/A	2a
Mendi et al	2023	Turkey	CT	76	36 (47.4%)	Manual (1 Label)	Olea Sphere 3.0 SP-21 (Radiomics)	LPAA 15,411,63 ± 17,552,36 Malignant/PHEO 56,164,18 ± 86,974,82	LPAA vs. PHEO/malignant	Logit-fit model: Accuracy 82.9% Sensitivity 84.2% Specificity 81.6% AUC 0.829	Positive	N/A	2a
Kim et al	2023	Korea	CT	308	156 (50.6%)	Manual (1 Label), Automatic	MEDIP U- Net	Test set: 4.6 ± 3.6 (R) 4.9 ± 4.0 (L)	Hyperplasia vs. normal	Segmentation DSC 0.7009 ICC 0.91 (95% CI 0.90–0.93) Classification Accuracy 0.948–0.961 Sensitivity 0.750–0.813 Specificity 0.973–1.000 AUC 0.98–0.99	Positive	N/A	2b
O’Shea A et al	2022	USA	CT	141	81 (57.4%)	Manual (1 Label)	3D Slicer PyRadiomics (Radiomics)	\	LPAA vs. adrenal metastasis	Predictive nomogram on the validation cohort: AUC 90.4% Harrell’s Concordance Index 0.9036 (95% CI 0.7180–1)	Positive	N/A	2b
De Leo et al	2022	Italy	CT	30	\	Manual (1 Label)	LIFEx (Radiomics)	\	PHEO characterization	Correlation with texture analysis: Urinary epinephrine/metanephrine levels (*R*^*2*^ = 0.946/*R *^*2*^ = 699) Ki-67 (*R*^*2*^ = 0.397) PASS score (*R*^*2*^ = 0.182) GAPP score (*R*^*2*^ = 0.705) Cellularity (*R*^*2*^ = 0.389)	Positive	N/A	2a
Xu et al	2021	China	CT	351 (18 adrenal lesions)	\	Unspecified	Universal Lesion Detector	\	Normal vs. abnormal	On external validation set: Sensitivity 33.3% PPV 85.7%	Limited	N/A	2a
Stanzione et al	2021	Italy	MRI	46	30 (65.2%)	Manual (1 Label)	ITK-SNAP PyRadiomics (Radiomics)	\	Benign vs. malignant	Accuracy 0.91 (95% CI 0.59–1.00) Sensitivity 0.91 PPV 0.92 F1 score 0.91 AUC 0.97 (95% CI 0.87–1.00)	Positive	N/A	2a
Moawad et al	2021	USA	CT	40	25 (62.5%)	Manual (1 Label)	Amira software PyRadiomics (Radiomics)	\	Benign vs. malignant	Sensitivity 84.2%, Specificity 71.4% AUC 0.85	Positive	N/A	2a
Luo et al	2021	China	CT	348	\	Manual (1 Label), Automatic	ITK-SNAP 3D U-Net Small-organNet	4.3 ± 2.1 (R) 5.7 ± 3.6 (L)	Conn, accurate segmentation	Segmentation (bilateral) DSC 87.42 ± 5.88 RVE 12.71 ± 13.83 HD95 2.89 ± 3.90	Positive	N/A	2b
Elmohr et al	2019	USA	CT	54	32 (59.3%)	Manual (1 Label)	Amira Software PyRadiomics (Radiomics)	\	Adenoma vs. ACC	Segmentation DSC 0.875 ± 0.04 Classification Accuracy 0.82 (95% CI 0.69–0.92) Sensitivity 0.81 Specificity 0.83 NPV 0.8 PPV 0.85 AUC 0.89	Positive	N/A	2a
Romeo et al	2018	Italy	MRI	60	\	Manual (1 spherical label)	ITK-SNAP 3D Slicer J48 classifier Weka v. 3.8.1 (Radiomics)	\	LRAA vs. LPAA vs. NAL	Accuracy 80% LRAA: AUC: 0.846 (95% CI 0.745–0.947) Sensitivity: 84% Specificity: 85% LPAA: AUC: 0.678 (95% CI 0.547–0.810) Sensitivity: 63% Specificity: 72% NAL: AUC: 0.860 (95% CI 0.767–0.952) Sensitivity: 89% Specificity: 82%	Positive	N/A	2a
Tang et al	2014	China	CT	10	\	Manual (Lesion and intratumoral necrosis), Automatic	Localized region-based level set method (LRLSM)	\	PHEO characterization	Arterial/portal venous phase: DSC 92.7%/92.9% TP 91.6%/92.5% FP 9.6%/7.8%	Positive	N/A	1
Saiprasad et al	2013	USA	CT	15	2 (13.3%)	Manual (1 Label), Automatic	\	\	Normal vs. abnormal	Sensitivity 80% Specificity 90%	Positive	N/A	1

*CT* Computed tomography, *MRI* Magnetic resonance imaging, *LPAA* Lipid-poor adrenal adenoma, *LRAA* Lipid-rich adrenal adenoma, *NAL* Non-adenoma adrenal lesions, *PHEO* Pheochromocytoma, *ACC* Adrenocortical carcinoma, *PA* Primary aldosteronism, *UPA* Unilateral primary aldosteronism, *BPA* Bilateral primary aldosteronism, *PASS* Pheochromocytoma of the Adrenal gland Scaled Score, *GAPP* Grading system for adrenal pheochromocytoma and paraganglioma, *R* Right, *L* Left, *1 Label* Adrenal gland/tumor in one segmentation. *DSC* Dice Similarity Coefficient, *RVE* Relative volume error, *HD95* 95th percentile of the Hausdorff Distance, *ICC* Intraclass correlation coefficient, *AUC* Area under the receiver operating curve, *IQR* Interquartile range, *CI* Confidence interval, *MLP* Multi-layer perceptron, *IOU* Intersection over union, *ASD* Average surface distance, *MAE* Mean average error, *RF* Random forest, *PPV* Positive predictive value, *NPV* Negative predictive value, *TP* True positive, *FP* False positive

**Table 2:** Detailed therapeutic study characteristics

Authors	Years	Country	Imaging modality	Patients	Female patients n (%)	Segmentation method	Software and models	Adrenal tumour volume (ml)	Detailed objective	Metrics	Outcome	Cost per 3D reconstruction	IDEAL stage
Sun et al	2023	China	CT	513	275 (53.6%)	Manual, possibly 2 Labels	3D slicer Python	\	Adrenalectomy risk assessment	On prospective set (RF model): Sensitivity 0.536 Specificity 0.820 Precision 0.484 AUC 0.724 F1 Score 0.508	Positive	N/A	2b
Du et al	2024	China	CT/MRI	32	5 (15.6%)	Semi-automatic (1 Label)	3DVAPS	113.31 ± 219.18	Ablation planning	Less insertion number (*P* = 0.035) Less complications rate (*P* = 0.029) Higher LRFS up to 5 years (*P* = 0.033)	Positive	N/A	2a
Wang Y et al	2023	China	CT	66 (of 119)	\	Unspecified, possibly 2 Labels	Commercial	\	Adrenalectomy risk assessment	Less HDI (*P* < 0.001)	Positive	N/A	2a
Yao et al	2022	China	CT	30	13 (43.3%)	Unspecified	Commercial	\	Surgical planning	Less operative time, blood loss (*P* < .001)	Positive	477,23 €	2a
Palomba et al	2022	Italy	CT	62 After PSM: 36	42 (67.7%) After PSM 21 (58.3%)	Unspecified, possibly 2 Labels	Commercial	\	Surgical planning	Less operative time, blood loss (*P* < .004)	Positive	N/A	2a
Zhao et al	2019	China	CT	49	\	Manual and automatic vessel segmentation	ITK-SNAP 3D U-Net	\	Adrenal tumour vessels segmentation	On test set: DSC 94.69% MIoU 90.22%	Positive	N/A	2a
Zhang et al	2018	China	CT	36	21 (58.3%)	Unspecified (2 Labels)	Commercial	\	Preoperative planning	Less operative time (*P* = 0.039) Better BP control: ΔSBP (*P* = 0.037) ΔDBP (*P* = 0.036)	Positive	N/A	2a
Wu S et al	2019	China	CT	12	2 (16.7%)	Unspecified, own software	3DVAPS	\	Ablation planning	Complete ablation 100% Local tumor control rates: 83.3% (1 year), 75.0% (2 and 3 years) Overall survival rates: 91.7% (1 year), 75.0% (2 years), 50.0% (3 years), 41.7% (4 years)	Positive	N/A	1
Souzaki et al	2015	Japan	CT	3	2 (66.7%)	Unspecified (1 Label)	Commercial	\	Preoperative planning	Complete resection 100% Complications 0%	Positive	N/A	1
Mitterberger et al	2006	Austria	CT	12	5 (41.7%)	Manual (2 Labels)	3DVIEWNIX	\	Preoperative planning (adrenal sparing)	Complete resection 100% Major complications 0%	Positive	N/A	1
Marvik R et al	2004	Norway	CT	5	\	Semi-automatic (not all labels specified)	CustusX (Navigation)	\	Surgical navigation	Mean registration accuracy 6.46 mm	Positive	N/A	1
Sebek et al	2022	Ireland	11C-metomidate PET/CT	14	\	Manual (1 Label), Semi-automatic for adjacent structures	\	1.249 ± 0.654	Ablation planning	Ablative dose to target 70% Adrenal sparing 83.5–96.4% Limited non-target thermal damage	Positive	N/A	0

*CT* Computed tomography, *MRI* Magnetic resonance imaging, *PET* Positron emission tomography, *PSM* Propensity score matching, *1 Label* adrenal gland/tumor in one segmentation, *2 Labels* adrenal tumor and adjacent gland labelled separately. *AUC* Area under the receiver operating curve, *RF* Random forest, *LRFS* Local recurrence‐free survival, *HDI* Intraoperative hemodynamic instability, *DSC* Dice similarity coefficient, *MIoU* Mean intersection over union, *SBP* Systolic blood pressure, *DBP* Diastolic blood pressure, Δ Difference between peak values during tumor handling and minimum values after pheochromocytoma/paraganglioma excision

Due to considerable heterogeneity in lesion types, diagnostic/therapeutic purposes, and the relatively recent adoption of volumetric 3D analyses, the data were unsuitable for meta-analysis. Therefore, a descriptive synthesis is presented. Eighteen studies (60%) targeted differential diagnosis, and 12 (40%) focused on treatment applications.

Outcomes were mainly reported as positive, integrating the performance metrics for lesion detection and classification, including automatic volumetric segmentation in diagnostic studies (Table 1), and adrenalectomy risk assessment, surgical and ablation planning, registration accuracy and clinical outcomes in therapeutic studies (Table 2). The chosen performance metrics for each included study varied according to the objective. Diagnostic studies consistently utilized metrics, such as sensitivity, specificity, accuracy, F1 score and area under the receiver operating curve (AUC). For studies that addressed automatization of segmentation, Dice Similarity Coefficient (DSC) was the principal reported evaluation criterion (Table 1). For therapeutic studies, the chosen metrics were dependent on the clinical relevance, ranging from assessment and reduction of surgical risks to ablation planning and execution (Table 2). According to the IDEAL framework, most studies were in early stages: one preclinical (stage 0; 3.3%), two first-in-human (stage I; 6.7%), and the majority (27/30, 90%) were developmental or exploratory (stage IIa–IIb). None reached randomized trials (stage III) or long-term monitoring and registry (stage IV).

### Risk of bias

QUADAS-2 quality assessment (Table 3) showed 17 of 30 studies (56.7%) had low risk of bias across all four domains. A high risk of patient selection bias was observed in 8 (26.7%) studies [31, 33, 34, 37–41] due to arbitrary or unspecified inclusion and lack of consecutive or random sampling. Four studies (13.3%) [32, 42–44] had unclear selection methods. Two studies (6.7%) showed unclear bias in the index test domain—one for using an unspecified 3D segmentation [41], and another for using a spherical volume of interest rather than a complete adrenal and lesion segmentation [31]. All studies had low bias in the reference standard domain. In the flow and timing domain, risk of bias was unclear in 9 studies (30%) [33–35, 37, 38, 40, 41, 43, 44], with the rest (21, 70%) deemed low risk.

**Table 3:**
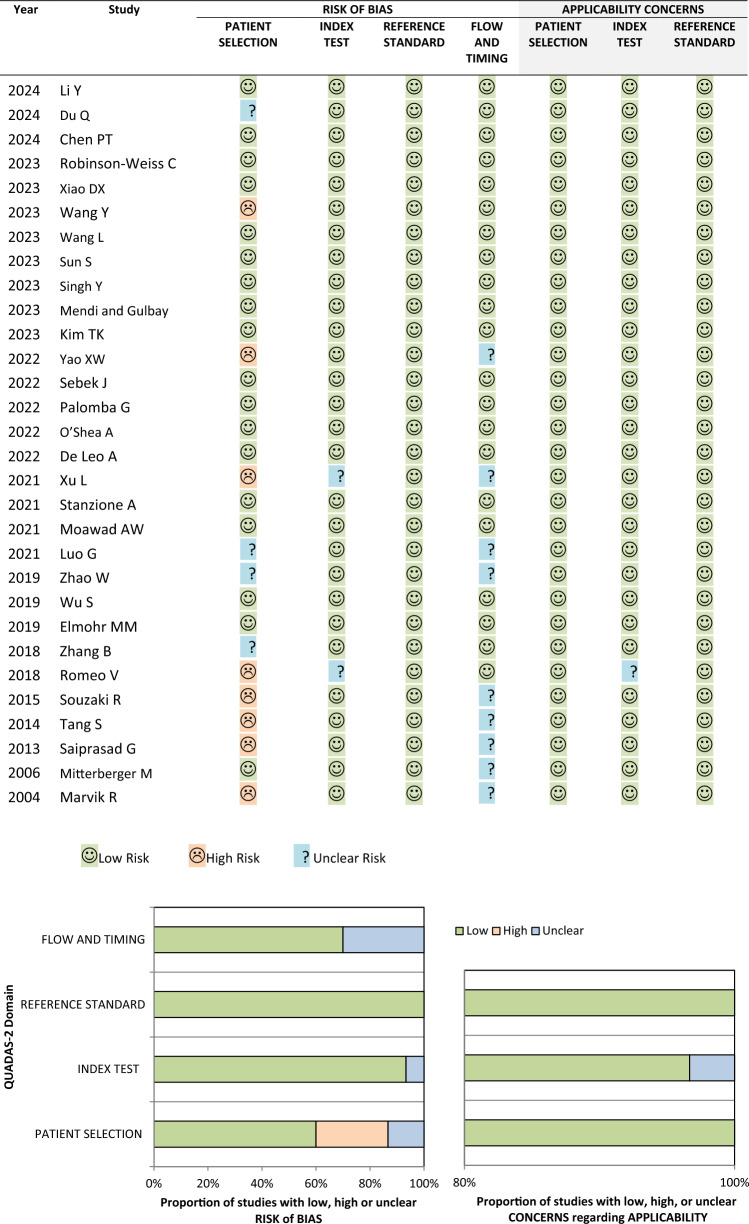
QUADAS-2 risk of bias graphical representation

### Segmentation approaches

Inclusion required volumetric 3D adrenal reconstruction; two were used with volume rendering [37, 40], and three reported 3D printing [37, 39, 40]. Adrenal segmentation was manual in 19 studies (63.3%), serving as the ground truth in 7/8 segmentation automatization studies [15, 33, 34, 43–46] (unspecified ground truth in [47]). One study applied spherical volumes of interest manually within lesions without segmenting the entire gland and lesion [31]. Semi-automatic adrenal segmentation was used in 3/30 studies [32, 38, 48], and in one for extra-adrenal structures [29]. Details were not specified in 8 studies, of which 5 used commercial, and 3 proprietary software for segmentation. Available annotation details revealed that in only 2 studies, separate labels were reported to differentiate ipsilateral normal adrenal parenchyma from neoplasia [35, 42], and were possibly used in 3 studies according to their figures [27, 28, 39] (total 5/29, 17.2%, as one study labelled vasculature). In most cases, one label was reported for adrenal gland/tumour (20/29, 69.0%), and 4 studies remained unclear regarding the different labels applied.

### Diagnostic applications

Adrenal gland and lesion volume calculations supported differential diagnosis of various neoplasms over the last decade. An automated multi-organ segmentation study assessing generalizability of a model trained on six public lesion data sets showed poor adrenal lesion detection sensitivity (33.3%), and missed homogeneous hyperplastic nodules [41]. Where reported, all groups used custom manual segmentations to build data sets for automation (unspecified ground truth in [47]). The most recent study trained a model on 885 CTs, and validated it on 280 external CTs with high segmentation accuracy (dice similarity coefficient: DSC 0.88 ± 0.06) and strong volume prediction consistency with manual methods (intraclass correlation coefficient: ICC 0.931, 95% CI 0.925–0.936); however, the data set contained twice as many normal adrenal glands when compared to hyperplasia and nodules (97–98% benign) [15]. Several other studies developed manual adrenal segmentations for automatic 3D segmentation with various algorithms. One study achieved DSCs of 0.70 (normal) and 0.71 (hyperplastic glands), with a high diagnostic performance to predict hyperplasia (area under the curve: AUC 0.99) [45]. Performance was higher for diffuse hyperplasia than for nodular cases, in which only surrounding parenchyma was identified. Sensitivity/Specificity in another study for automatic segmentation and differentiation between normal adrenal glands and lesions was 69%/91% across a test set with 991 CTs [46]. A model for adrenal metastases segmentation in CT scans of 182 patients outperformed earlier methods (mean DSC: 0.86) [47], while another used several algorithms on a primary hyperaldosteronism data set to increase segmentation accuracy [44]. Manual segmentations of pheochromocytomas and necrosis yielded automated DSCs of 0.88–0.96 in arterial and portal venous phase CTs [33]. Automated identification and histogram analyses based on manual segmentations of 30 adrenal glands versus background achieved 80% sensitivity and 90% specificity for adrenal abnormality detection [34].

Ten studies (33.3%) incorporated 3D radiomic and texture analysis between 2018 and 2024. Radiomics from CT [49] and MRI [30] helped distinguish benign from malignant lesions, including lipid-poor adenomas (LPAs) vs. subclinical pheochromocytomas [48], adrenocortical carcinoma [50] and lipid-rich adenomas [31, 51, 52]. Models addressed specific contexts, such as primary hyperaldosteronism [53], pheochromocytoma [54], and differentiation of large adenomas from carcinomas [55]. RQS assessment [26] (Table 4) shows a median total score of 3.5 (IQR: 0.3–10.3), ranging from − 3/36 (0%) to 13/36 (36%). The median relative percentage score was 9.7% (IQR: 0.7–28.6%). All but one study (90%) documented imaging protocols, 100% reported discrimination statistics, 80% calibration statistics. However, 70% lacked external validation, none were prospective, none tested robustness to temporal variabilities or performed phantom analyses, highlighting common limitations in reproducibility and generalizability, and none reported cost-effectiveness.

**Table 4:** Radiomics quality score (RQS) assessment as published by Lambin et al. 2017 for all included articles, calculating individual scores per item converted to a percentage

Study	Item 1	Item 2	Item 3	Item 4	Item 5	Item 6	Item 7	Item 8	Item 9	Item 10	Item 11	Item 12	Item 13	Item 14	Item 15	Item 16	Total	
Chen PT et al. 2024	1	0	0	0	−3	1	0	0	2	1	0	−5	0	0	0	1	−2	0%
Xiao DX et al. 2023	1	1	0	0	3	0	0	1	1	1	0	4	0	0	0	1	13	36%
Singh Y et al. 2023	0	0	0	0	3	0	0	0	2	1	0	−5	0	0	0	0	1	3%
Mendi BAR et al. 2023	1	1	0	0	3	0	0	0	2	1	0	−5	0	0	0	0	3	8%
O’Shea A et al. 2022	1	0	0	0	3	0	0	0	2	1	0	−5	0	0	0	1	5	14%
De Leo A et al. 2022	1	0	0	0	3	1	0	0	1	1	0	−5	0	0	0	1	−3	0%
Stanzione A et al. 2021	1	1	0	0	3	0	0	0	2	1	0	−5	0	0	0	1	4	11%
Moawad AW et al. 2021	1	0	0	0	3	0	0	0	2	1	0	−5	0	0	0	1	3	8%
Elmohr MM et al. 2019	1	1	0	0	3	0	0	0	2	0	0	2	2	0	0	0	11	31%
Romeo V et al. 2018	1	0	0	0	3	0	0	0	2	0	0	2	2	0	0	0	10	28%

### Therapeutic applications

Nine studies investigated 3D image guidance for adrenalectomy [27, 28, 35, 37–40, 42, 43], including one prospective study [27]; three addressed ablation therapies [29, 32, 36].

Early augmented reality applications in laparoscopic adrenalectomy showed promise: one pilot study registering 3D preoperative imaging to the tracked laparoscope view—with 45min processing time per case and 6.9mm registration accuracy—reported increased procedural safety in 5 out of 6 cases due to enhanced information during dissection, and Doppler-assisted blood vessel identification [38]. Subsequent studies reported that 3D reconstruction enhanced safety in image-guided adrenalectomy by significantly reducing operating time and blood loss, as well as blood pressure fluctuations in pheochromocytoma [27, 35, 42]. One emphasised the advantage of SR over VR images for differentiating vasculature, neoplasia and normal adrenal tissue, facilitating minimally invasive cortical-sparing surgery for Conn adenomas and pheochromocytomas [35]. Addressing the adrenal glands’ abundant arterial supply, one group created a manually annotated CT angiography data set and achieved digital subtraction angiography-like automatic vessel segmentation (DSC 0.95 in 304ms), supporting surgical planning and vascular preservation [43].

In paediatric adrenal neuroblastoma (*n* = 3, age 1–20 months, weight 4.1–10.4 kg), 3D-printed models based on individual volumetric reconstructions informed optimal port placement, facilitating laparoscopic procedures [37]. A retrospective comparison of open surgical outcomes for large pheochromocytomas and paragangliomas without and with preoperative virtual and 3D-printed models (*n* = 15 per group, tumour diameter 17.8 ± 4.4 vs. 18.3 ± 5.6), found improved vascular handling and significantly reduced of operating time and blood loss in favour of guidance with 3D-printed models, with R0 resections in all [40]. Similarly, 3D printing was identified as an independent protective factor against haemodynamic instability in minimally invasive phaeochromocytoma surgery (*n* = 119) [39]. Machine learning models integrating clinical and radiomic data also showed potential to predict adrenalectomy difficulty (*n* = 396 training/validation; *n* = 117 testing) [28].

Only 3 studies explored 3D-assisted adrenal ablation. One simulated microwave ablation thermal profiles for 14 aldosterone-producing adenomas, using biophysical modelling to optimise applicator placement while limiting thermal damage to adjacent structures and normal adrenal parenchyma [29]. In a pilot cohort undergoing transhepatic ultrasound-guided percutaneous microwave ablation for right-sided adrenal metastases, 3D CT planning enabled complete ablation in 10/12 (83.3%) in one session, and in 2/12 (16.7%) in two, with fewer applicators, insertions, and ablation points, and optimised placement of multiple applicators near critical structures, with 3 recurrences during median follow‐up of 31 months (range 6–52) [36]. A retrospective comparison found that 3D planning (*n* = 32) for ultrasound‐guided percutaneous microwave ablation of predominantly metastatic malignant adrenal tumours led to significantly fewer applicator insertions, overall complications, and local progression than 2D planning (*n* = 30), especially for lesions ≥ 5 cm, during median follow-up of 30 months (range 3–84) [32].

## Discussion

This systematic review synthesizes current evidence on the role of 3D image guidance in managing adrenal disease, assessing its diagnostic and therapeutic applications.

In diagnostics, recent studies apply volumetric reconstructions and segmentation masks to assess adrenal volume, a proxy for functional parenchyma in various endocrine disorders. Unlike two-dimensional CT slices, 3D reconstructions enable full lesion assessment within anatomical context, including tumour margins, internal heterogeneity, and adjacent normal adrenal parenchyma. Manual annotations remain essential for both initial reconstructions and development/validation of (semi-)automatic segmentation tools. However, adrenal glands’ variability in shape and proximity to other organs complicates manual delineations, leading to inconsistencies, and interobserver variability [15].

Our assessment of the broad spectrum of 3D reconstruction use in diagnosis and treatment of adrenal disease revealed that there is an increasing number of studies developing (semi-) automatic 3D segmentations to overcome the limitations of time-consuming manual annotations requiring expert clinical knowledge. These studies focus on simplified subgroup analyses (e.g., normal vs. abnormal, normal vs. hyperplasia, normal vs. metastasis, Conn adenomas, pheochromocytomas), limiting their utility for meta-analysis and failing to represent the spectrum of adrenal diseases requiring surgery. In addition, segmentations often fail to differentiate between normal preservable parenchyma and neoplastic areas, labelling the entire adrenal zone with only one segmentation. Only 17.2% of included studies showed two labels to differentiate adrenal tumours from ipsilateral normal parenchyma. Simplified ground truths (single labels per side or for both) can cause false negatives in lesion detection [45]. Of note, many excluded studies trained models only on healthy adrenal glands, which are over-represented in open-source data sets.

**Fig. 2:**
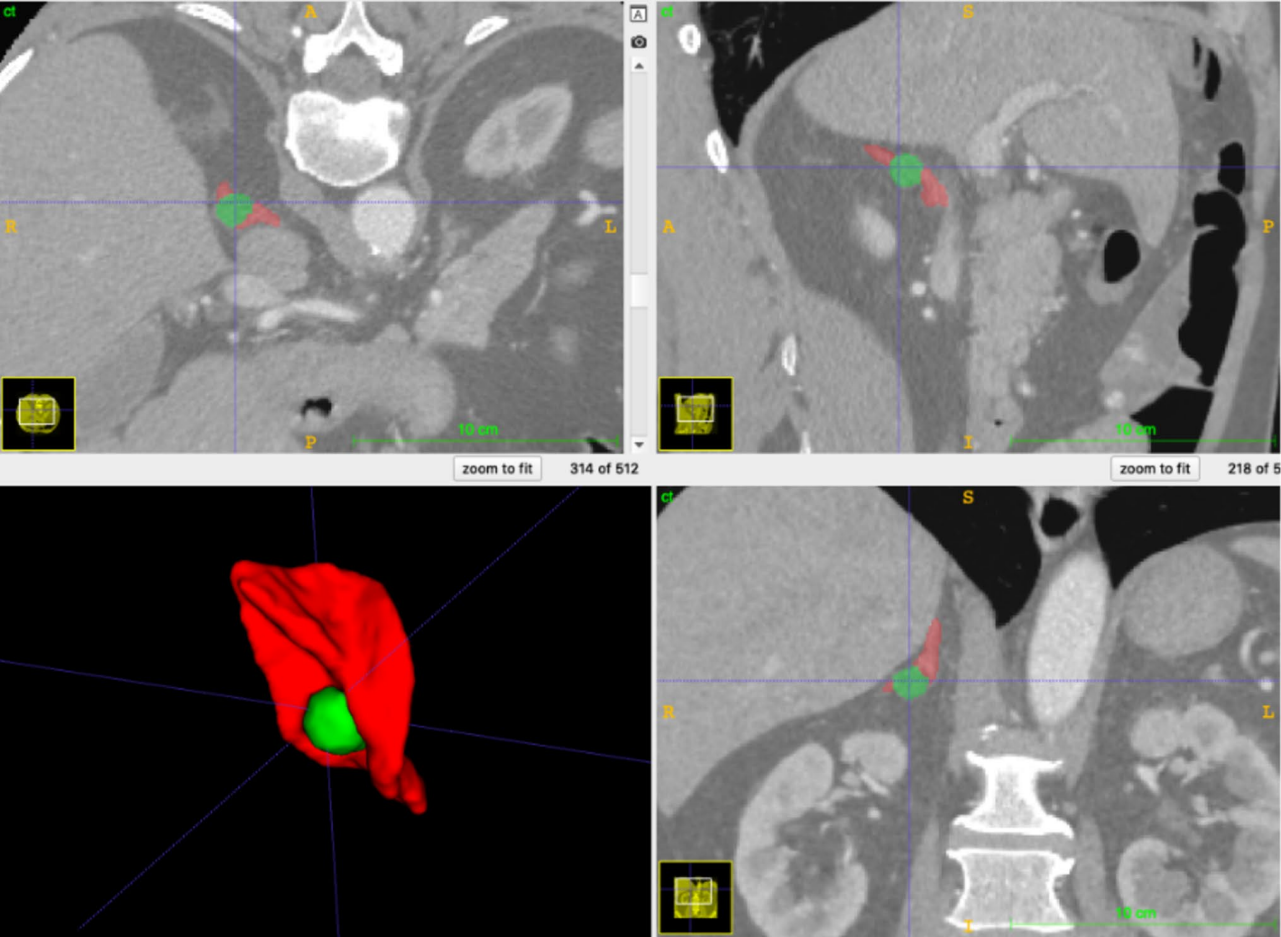
Volumetric 3D segmentation with precise separation of normal parenchyma (red) and tumour (green) in all three dimensions of an exemplary CT scan from the authors’ own database, to illustrate the potential for cortical-sparing approaches based on differentiated image analyses (Color figure online)

Despite the rise in adrenal gland segmentation models, limited generalizability still hinders broad clinical applicability [15]. Considering suboptimal model performances across the full spectrum of adrenal disease, the present analysis underscores the need for dedicated and representative annotated data sets. A data set distinguishing neoplastic from healthy parenchyma (Fig. 2) would have high clinical relevance for reliable segmentation and classification, enabling not just lesion volume estimation, but also assessment of remnant parenchyma suitable for partial adrenalectomy.

Radiomics, beginning with 3D segmentation, analyzes pixel/voxel patterns and spatial relationships to extract quantitative imaging features. This non-invasive diagnostic tool was applied across oncology [17, 26], and 10 studies used it for adrenal lesion characterisation [30, 31, 48–55], mostly using 3D texture analysis. It has shown particular value for radiologically indeterminate, non-functioning lesions, for example, to distinguish lipid-poor adenomas from malignant tumours and subclinical pheochromocytomas [48, 50, 52].

Despite its promise, adrenal radiomics remains methodologically inconsistent. Over 60% of studies lacked external validation and demonstrated poor calibration statistics. None were prospective or evaluated cost-effectiveness. Even within radiomics in oncology, groups using the Radiomics Quality Score (RQS) identified inconsistencies and lack of standardized evaluation of scientific rigor and clinical relevance, even in high-impact publications [26, 56, 57]. Our systematic review revealed similar shortcomings in adrenal radiomics, with low median and maximum RQS scores, highlighting the need for improved credibility and utility in precision adrenal diagnostics.

Therapeutically, most studies (75%) focused on image-guided surgery, and 25% addressed probe-based ablation. 3D virtual models clarified spatial relationships between adrenal glands, vasculature, and tumours, and aided in anticipation of surgical challenges. As early as 2004, 3D navigation improved anatomical orientation and vascular localization during adrenalectomy through interactive, multi-angle visualization [38], just like intraoperative augmented reality overlay [18]. Preoperative vascular segmentations helped identify anatomical variants, potentially reducing vessel injury and preserving function [43].

Adrenal surgery is complex, with potential for serious complications [58]. Given the volume–outcome relationship and relative procedure rarity, European guidelines recommend a minimum of six adrenal surgeries per surgeon annually, and 20 for adrenocortical carcinoma [59, 60]. Yet only a third of surgeons meet these thresholds, performing just over half of all adrenalectomies [61]. Most perform fewer than 6 annually, requiring extended periods to achieve full procedural proficiency, if ever [62, 63]. In this context, 3D reconstructions enhanced overall surgical safety and efficiency, reducing operative times and blood loss. They are particularly useful in complex cortical-sparing and oncologic resections, supporting surgical simulation and calculation of remnant adrenal volume [35].

Three studies reported 3D printing as valuable for preoperative planning and operative guidance [37, 39, 40]. Numerous excluded case reports underscore interest in physical models that support planning and training, though further trials are needed.

We also identified early applications of 3D reconstructions for adrenal tumour ablation—a technique well-established in liver interventions [64]. Adrenal tumour ablation is challenging due to their proximity to critical structures. The three studies that applied 3D visualization reported enhanced tumour understanding, improved ablation guidance, safety and efficacy [29, 32, 36]. Given not only the challenges of automatic segmentation for pre-procedural imaging, but also for the intraprocedural dynamic environment with deformations due to applicator insertion [29], precise registration during such navigation approaches remains a key challenge that may be resolved by fusion with real-time interventional imaging techniques like ultrasound.

Overall, the available literature reflects an increasing interest in 3D image guidance for diagnosis and treatment of adrenal disease but remains in the early stages of innovation. Current evidence does not support making 3D image reconstructions mandatory for the diagnosis or treatment of adrenal disease. However, growing interest in their role in differentiating adrenal neoplasms, along with improved accessibility and automated reconstruction techniques that may reduce costs, highlights their potential utility in enhancing surgical planning, particularly in complex cases. Potential applications go beyond surgical decision-making, also enhancing surgical education and training, as well as patient counselling. However, available data do not support a comprehensive analysis of the health-economic impact of 3D reconstructions for surgical guidance in adrenal disease.

In the future, clinical effectiveness evaluation studies need to be complemented by context-specific cost-effectiveness and implementation research, particularly in resource-limited settings, where feasible, sustainable implementation models are most needed.

## Conclusion

Adrenal disease management via 3D imaging is a promising and evolving field, lagging behind other oncologic domains due to its rarity and the need for large-scale well-annotated data sets to make the best use of the increasingly available computing resources and machine learning tools for segmentation automatization and quantitative image analyses. Integration of multiple modalities—such as the combination of 3D imaging with laparoscopes or miniaturised ultrasound probes—provides promising future avenues for surgical navigation.

Current evidence supports the role of 3D imaging in enhancing lesion delineation and characterisation, enabling image-based decision support in the pre- and intraprocedural stages to facilitate safer treatment strategies. However, widespread adoption of radiomics and automated segmentation in clinical practice needs continued efforts in methodological rigor, data set diversity, and external validation. As clinical validation expands and long-term outcomes are studied, 3D image guidance may become a cornerstone of precision medicine in adrenal disease.

## Data Availability

All data generated or analysed during this study are included in this published article.
